# From Discovery to Justification: Outline of an Ideal Research Program in Empirical Psychology

**DOI:** 10.3389/fpsyg.2017.01847

**Published:** 2017-10-27

**Authors:** Erich H. Witte, Frank Zenker

**Affiliations:** ^1^Social and Economic Psychology, University of Hamburg, Hamburg, Germany; ^2^Philosophy and Cognitive Science, Lund University, Lund, Sweden; ^3^Institute of Philosophy, Slovak Academy of Sciences (SAS), Bratislava, Slovakia; ^4^Philosophy, Konstanz University, Konstanz, Germany; ^5^Institute of Logic and Cognition, Sun Yat-sen University, Guangzhou, China

**Keywords:** confirmation, knowledge accumulation, meta-analysis, psi-hypothesis, replicability crisis, research programs, significance-test, test-power

## Abstract

The gold standard for an empirical science is the replicability of its research results. But the estimated average replicability rate of key-effects that top-tier psychology journals report falls between 36 and 39% (objective vs. subjective rate; Open Science Collaboration, 2015). So the standard mode of applying null-hypothesis significance testing (NHST) fails to adequately separate stable from random effects. Therefore, NHST does not fully convince as a statistical inference strategy. We argue that the replicability crisis is “home-made” because more sophisticated strategies can deliver results the successful replication of which is sufficiently probable. Thus, we can overcome the replicability crisis by integrating empirical results into genuine research programs. Instead of continuing to narrowly evaluate only the stability of data against random fluctuations (*discovery context*), such programs evaluate rival hypotheses against stable data (*justification context*).

## Introduction

Empirical psychology and the social sciences at large remain in crisis today, because (too) many key-results cannot be replicated (Baker, 2015; Open Science Collaboration, 2015; Etz and Vandekerckhove, 2016). Having diagnosed a disciplinary crisis as early as Willy (1889), psychologists did so most recently in a special issue of *Perspectives on Psychological Science* (Witte, 1996a; Pashler and Wagenmakers, 2012; Spellman, 2012; Sturm and Mülberger, 2012). Particularly the crisis of significance-testing is about as old as the test itself (Witte, 1980; Cowles, 1989; Harlow et al., 1997). Setting the current crisis apart is the insight that null-hypothesis significance testing (NHST) has broadly failed to deliver the stable effects that should characterize empirical knowledge. Many researchers are therefore (rightly) concerned that *all* published effects are under doubt. The perhaps most pressing question today is how our field might regain trust.^1^

In our view, the ongoing replicability crisis reflects a goal-conflict between publishing statistically significant results as an individual researcher and increasing the trustworthiness of scientific knowledge as a community (Bakker et al., 2012; Nosek et al., 2012; Ioannidis, 2014). Acknowledging that we can separate the corresponding research activities only analytically, we map both goals onto the terms ‘discovery’ and ‘justification’ (aka ‘DJ-distinction’). Since the *status quo* favors “making discoveries,” we submit, the balance between these goals must be redressed. As regards variously proposed “minimally invasive” remedies, however, we find that such “soft” measures are insufficient to regain trust.^2^

We rest our case on the observation that psychologists typically deploy statistical inference methods in underpowered studies (Maxwell, 2004). This praxis generates theoretically disconnected “one-off” discoveries whose replication is improbable. But such results *should* not be trusted, because they are insufficiently stable to justify, or corroborate, a theoretical hypothesis (Rosnow and Rosenthal, 1989). By contrast, corroboration *is* possible within a research program. To overcome the crisis, therefore, our community should come to coordinate itself on *joint* long-term research endeavors.

## From Discovery to Justification

### Overview

Constructing a psychological theory begins with discovering non-random relations between antecedent variables and their (causal) consequences, aka *stable* effects. Relying on a probabilistic version of the lean DJ-distinction, this section contrasts the discovery and the justification context, explains the replicability of empirical results, and shows why underpowered discoveries cannot be trusted. We then define two key concepts: *induction quality of data* and *corroboration quality of hypotheses*, and formulate a brief upshot.

### Stable Effects

As late 19th-century psychologists transformed their field into an empirical science, the guiding idea was to base empirical hypotheses on stable non-random effects. Indeed, only stable effects guide researchers toward the *explananda*. Otherwise, explanation would be pointless—for what should be explained? This pedestrian insight makes the discovery of a stable effect as necessary as the managing of random influences is regularly unavoidable (e.g., in measurement, sampling, or situation-construction). We therefore discover an effect but in a *probabilistic* sense.

As we thus evaluate our chances of having made a discovery, we must gauge the effect’s deviation from random against a statistical significance threshold. Of course, if we cannot discover an effect with certainty, then it follows by parity of reasoning that we cannot falsify it with certainty either. We must therefore ever invest *some* trust that the effect in fact surpasses potential random influences.

Also known as ‘stable observations,’ such effects register as highly probable deviations from a content-free random or null-hypothesis (H_0_). But even a stable effect (in this probabilistic sense) may be subsumed under distinct explanatory hypotheses. We must therefore corroborate such diverging explanations via a theory that predicts the effect’s probabilistic signature from initial and boundary conditions.

If we base hypothesis construction and validation on uncertain observation, then hypothesis corroboration likewise entails the probabilistic comparison of a hypotheses pair. *Pace* efforts by the likes of Rudolf Carnap and Karl Popper, this is an immediate consequence of recognizing that uncontrollable random influences are relevant to theory acceptance. As we now show, it is this interplay between an effect’s probabilistic discovery and its probabilistic corroboration that connects the discovery and the justification contexts (aka DJ-distinction).

### The Lean DJ-Distinction

Discovery and justification are fairly self-evident concepts. The dominant mode of deploying statistical inference methods nevertheless reflects them inadequately. Indeed, a review of the textbook literature would show that the NHST approach largely ignores the DJ-distinction. To set this right, we rely on a probabilistic model to make a non-controversial version of the DJ-distinction precise.^3^ Hoyningen-Huene (2006) calls it the ‘lean DJ-distinction’ to denote “an abstract distinction between the factual […] and the normative or evaluative […]” (ibid., p. 128).

In adapting this distinction, ‘discovery’ refers to research activities in the *data space* that employ probabilities, while ‘justification’ denotes activities in the *hypotheses space* that employ likelihoods. Unlike a measure of the probability, *P*, of data given a hypothesis [where 0 ≤*P*(D,H) ≤ 1], likelihoods, *L*, aka ‘inverse probabilities,’ are a sort of probability measure for hypotheses given data [where 0 < *L*(H|D) < ∞]. ‘Data space’ and ‘hypotheses space’ are analytical constructs, respectively denoting the collection of stable data and their subsumption under hypotheses.

Of course, research activities alternate between both spaces, witness metaphorical ideas, and such practices as data-“torturing” or inventing *ad hoc* hypotheses, etc. Similar heuristics are fine, but they do not amount to a hypothesis test. After all, discovery context activities focus on phenomena we are yet to discover. So a probabilistic model defines a discovery as a (theory-laden) observation of a non-random effect. This yields as relevant elements the H_0_-hypothesis and the actual data distribution. If data have a low probability given the H_0_ (aka the effect’s *p*-value; see Fisher, 1956), this is called a ‘discovered effect.’

But when we next evaluate the *trustworthiness* of data, the crucial question is this: if we treat the data distribution that had been obtained in a given test-condition as a theoretical parameter, and moreover hold constant the *p*-value and the number of observations, what is the probability of obtaining the *same* distribution given the H_0_ in a subsequent instance of this test-condition?^4^ So to assess the trustworthiness of data *is* to measure the probability of replicating a non-random effect. This leads us to consider test-power.

### Test-Power and Statistical Significance vs. Theoretical Importance

Cohen (1962) had suggested that empirical studies in the social sciences tend to be underpowered. This entails a *low* probability of successfully replicating a non-random effect. His guiding assumption was that the true amount of influence from independent onto dependent variables be of medium size (*d* = 0.50).

Provided a typical sample size around n1 = n2 = 30, if we assume *d* = 0.50 and a two-sided α-error = 0.05, then average test-power comes to 1–β-error = 0.46. Some 25 years later, Sedlmeier and Gigerenzer (1989) arrived at a similar value. (The α-error denotes the chance of obtaining a false positive test-result, the β-error the chance of obtaining a false negative result; both errors are normally non-zero, and should be small for data to be trustworthy.) Test-power = 0.46 implies that samples are typically too small to expect a *stable* effect. Therefore, empirical results obtained with similar test-power may at best issue invitations to more closely study such “discoveries.”

In psychology as elsewhere in the social sciences, however, researchers tend to over-report such underpowered results as hypothesis *confirmations*. Already some 50 years ago, this praxis was identified as a cause of publication bias (Sterling, 1959). To more fully appreciate why this overstates the capabilities of the method applied, consider that NHST does normally not specify the H_1_. One thus fails to assign precise semantic content to it. By contrast, the (random) H_0_ does tend to be well-specified.^5^

If data now display a sufficiently large deviation from random, this is (erroneously) interpreted as a discovery of theoretical importance. But on the reasonable assumption that theoretically important discoveries are stable rather than random, a *necessary* condition in order to meaningfully speak of a theoretically important discovery is to have properly employed a reproducibility measure such as test-power. This in turn presupposes a specified effect size, which the standard mode of deploying NHST, however, cannot offer. So NHST cannot warrant an immediate transition from ‘statistically significant effect’ to ‘theoretically important discovery.’

Many researchers appear to be satisfied knowing that test-power is maximal, but fail to check its exact value. So they do not *properly* deploy a reproducibility measure. The one “good” reason for this is their failure to specify the H_1_, because only its specification renders test-power quantifiable. This failure contributes to the confidence loss in our research results. Understandably so, too, for some 50 years after Cohen had hinted at *d* = 0.46, test-power typically registers even lower, at *d* = 0.35. So *most* studies are underpowered (Bakker et al., 2012; Baker, 2015).

Against the background of our probabilistic model, we proceed to explain why so many empirical results should not be trusted.

### Trustworthy Discoveries

In his influential textbook Cohen had recommended that “[w]hen the investigator has no other basis for setting the desired power value, the value [1–β-error = 0.80] is used” (Cohen, 1977, p. 56). This set a widely accepted standard. Together with α = 0.05, it implies a weighing of epistemological values that makes the preliminarily discovery of an effect *four* times more important than its stable replication. (This alone goes some way toward explaining 1–β-error = 0.35.) Similarly-powered “discoveries” thus are typically instable.

It follows that few effects which arise in the discovery context are *known* to deserve a theoretical explanation. Hence, the unsophisticated application of NHST as a discovery method regularly fails to inform the evaluation of hypotheses in the justification context. Since justification contexts activities aim at developing the comparatively best-corroborated hypotheses into theories, it can hardly surprise that psychology offers so few genuine theories (we return to this in Section “Precise Theoretical Constructs?”).

To set this right, discovery context activities must establish non-random effects that also feature a high replication probability, i.e., *stable* effects. Yet, the current research- and publication-praxis does not fully reflect that insight. For instance, though “mere” replications do serve to evaluate whether an effect is stable, until recently one could not publish such work in a top-tier journal (see Holcombe, 2016 on *Perspectives on Psychological Science*’s new replication section).

In summary, we can quantify the replication probability of data only if we specify *two* point hypotheses (H_0_, H_1_). To improve the trustworthiness of empirical knowledge, we must therefore increase the precision of theoretical assumptions (Klein, 2014). For only this yields knowledge of test-power, and only then has the comparative corroboration of the H_1_ by a data-set D (as compared to a rival H_0_) *not* been indirectly deduced from our estimation of D given the H_0_.

This puts us in a position to offer two central definitions.

### Induction Quality of Data, Corroboration Quality of Hypotheses

Our probabilified version of the lean DJ-distinction suggests that two analytically distinct activities govern the research process. Roughly, one first creates an empirical set-up serving as a test-condition to obtain data of sufficient induction quality (discovery context). Next, one tests point-hypotheses against such data (justification context).

In more detail, discovery context activities evaluate data by means of descriptive and inferential statistics given fixed hypotheses. Since this gauges *induction quality of data*, we perform an evaluation in the data space. (In fact, we proceed hypothetically, effectively assessing if data would be sufficiently trustworthy if they were obtained.) Exactly this is expressed by ‘gauging the probability that data are replicable given two point-hypotheses.’

Proceeding to the justification context, we now evaluate point-hypotheses in order to gauge *corroboration quality of hypotheses* given data. So we perform an evaluation in the hypotheses space. Crucially, only if we in fact obtain data of sufficient induction quality can we properly quantify the inductive support that actual data lend to a hypothesis. So ‘gauging corroboration quality’ refers to evaluating the degree to which probably replicable data support one hypothesis more than another.

Before, we apply these distinctions in the next section, we can define as follows:

*Def. induction quality*: A measure of the sensitivity of an empirical set-up (given two specified point-hypotheses and a fixed sample size) that is stated as α- and β-error. Though a set-up’s acceptability rests on convention, equating both errors (α = β) avoids a bias *pro* detection (α-error) and *con* replicability (β-error). Currently, α = β = 0.05 or α = β = 0.01 are common standards. Based on Neyman–Pearson theory, this measure is restricted to the discovery context; it qualifies the test-condition itself. Since we can gauge induction quality *without* actual data, this has nothing to do with a hypothesis test.
*Def. corroboration quality*: A comparative measure of the inductive support that data lend to hypotheses, stated as the likelihood-ratio (aka ‘Bayes-factor’) of two point-hypotheses given data of sufficient induction quality. The support threshold is the ratio (1–β-error)/α-error, and so depends on induction quality of data. For instance, setting α = β = 0.05 yields a threshold of 19 (or log 19 = 1.28), and α = β = 0.01 yields 99 (log 99 = 2.00), etc. Based on Wald’s non-sequential testing theory the measure tests hypotheses against *actual* data in the justification context (Azzalini, 1996; Royall, 1997).

Of course, the final letter in NHST continues to abbreviate the term ‘test.’ After all, NHST does test the *probability* of data given a hypothesis, *P*(D,H). But our definitions imply that data of low replication probability are insufficient to test a hypothesis in the sense of gauging its *likelihood, L*(H|D). This is because a low-powered “discovery” of effect E in test-condition C (as indicated by a large β-error) entails the *improbability* of redetecting E in subsequent instances of C. So even in view of a confirmatory likelihood-ratio, data of insufficient induction quality may well-initially support a hypothesis. But similar support need not arise in new data of sufficient induction quality. So a given hypothesis may subsequently fail to be corroborated.

### Upshot

On this background, the ongoing debates between statistical “schools” seem to be academic ones. After all, most extant estimation procedures for statistical significance operate squarely in the data space (Royall, 1997; Gelman, 2011; Wetzels et al., 2011). But if future theoretical developments must come to rely on coordinated activities that integrate the data with the hypotheses space, then the current crisis of empirical psychology would (at least partially) have arisen as a consequence of a methodologically *unsound* transition from discovery to justification. A sound version thereof, as we saw, leads from stable effects to trustworthy discoveries and on to acceptable forms of hypothesis corroboration.

As we also saw, integrating both spaces presupposes that we specify the expected empirical observation as a point-value. This states a theoretically sound minimum effect size (whether derived from a theory, or not); in uncertain cases it states a two-point interval placed around that value. By contrast, all alternative strategies simply let data have the “last word” on how we should construct a data-saving hypothesis. But this runs directly into the unmet challenge of validating induction.

Indeed, the risk of being “perfectly wrong” should be accepted even for precise theoretical assumptions. After all, being “broadly right” under merely vague assumptions is to accept virtually all non-random data-saving hypotheses. But that obviously fails to inform theoretical knowledge.

## Case Study: Psi-Research

### Overview

To clarify the relation between hypothesis corroboration and data replication, we exemplify our distinctions with a fairly controversial effect, treat its size, point to future research needs, and summarize the main insight.

### Replicating Bem’s Psi-hypothesis

Bem’s (2011) infamous results on precognition allegedly support the hypothesis that future expectations influence present behavior (aka ‘psi-hypothesis’). Seeking to replicate Bem’s data, Wagenmakers et al. (2012) claimed to pursue a confirmatory research agenda. They could stop inquiry after 200 sessions with 100 subjects (see their Figure 2; ibid., p. 636). For by then their data had lent 6.2 times more support to the H_0_ (read: *no* influence from future expectations) than to the H_1_ (read: influence). This is considered *substantial* evidence for the H_0_ (Jeffrey, 1961; Wagenmakers et al., 2011). Since Wagenmakers and colleagues base their inquiry on Wald’s (1947) sequential analysis, however, we see reasons to treat their result with caution.

Rather than in order to evaluate theoretical hypotheses, Wald (1947) had developed sequential testing during WWII as a quality-control method in ammunition production. Its immediate purpose was to estimate how many shells in a lot deviate from a margin of error, M. While any deviation exceeding M provides a sufficient reason to discard the whole lot, measuring large deviations from M is of course less cumbersome than measuring small ones. So it saves effort to infer *probable but unobserved* small deviations from large observed deviations.

Wald’s sequential testing strategy provides a rather brilliant solution to the classical problem of inducing properties of the whole from its parts. But it cannot generalize to *additional* lots. Nor was it intended to induce over abstract categories, but rather over material objects. Applied to the case of Wagenmakers and colleagues testing the specified hypotheses *p* = 0.50 vs. *p* = 0.531 (based on Bem’s 2011 first experiment), this means that neither the α- nor the β-error were known. After all, the number of observations keeps varying with the observed result. Without at least stipulating both errors, however, we cannot quantify the replicability of their result. So we should rather not trust it.

To explain, our previous section had shown that hypothesis testing requires trustworthy data. To more fully appreciate that trustworthiness is largely owed to knowledge of errors (Mayo, 1996, 2011), recall that induction quality and corroboration quality are related: if induction quality is unknown, then corroboration quality remains diffuse, and so can at best facilitate a vague form of justification. After all, even if a new and larger sample includes “old” data, a subsequent sample may nevertheless lead to a contrary decision as to whether a hypothesis is confirmed, or not.

As Rouder’s (2014) discussion of the stopping rule shows (nicely), it is for this reason that Bayesians recommend that we *keep* adjusting our confidence level as data come in. Indeed, Bayesian inference puts the “focus on the [*current*] degree of belief for considered models [here: H_1_ and H_0_], which need not and should not be calibrated relative to some hypothetical truth” (ibid., p. 308). Hence, the authors could reject Bem’s hypothesis (H_1_), and instead accept the H_0_ at a confidence level of (1–α-error)/β-error = 1.64, where 1–α = 0.95 and β = 0.58.

But the matter is more intricate yet. After all, corroboration quality would change if we altered the presumed distribution of *possible* data (aka ‘the priors’), for instance from a Cauchy- to a normal-distribution (Bem et al., 2011). Consequently, the *same* actual data would now rather support the alternative hypothesis. In general, which hypothesis it is that data confirm can be manipulated—intentionally or not—by suitably selecting the distribution of possible data. So “that different priors result in different Bayes factors should [indeed] not come as a surprise” (Ly et al., 2016, p. 12).

The selected type of prior distribution, however, is logically independent of the hypothesis we wish to test, ever entails weighing one hypothesis against another, and ultimately reflects a subjective decision. This provides reasons against giving Bayes-factors *alone* the final say in hypothesis corroboration. By contrast, to point-specify the hypothesis one does test eliminates this caveat by avoiding the priors, and no other method does. In the absence of a specified H_1_, then, whether a test-condition suffices for a clear justification of the H_0_ depends on it featuring acceptably low α- and β-errors.

It follows that, had Wagenmakers and colleagues stopped their test after a mere 38 sessions, the same sequential testing-strategy should have led them to accept the H_1_ on the basis of *nearly substantial* evidence (a likelihood-ratio of 3). This would have “confirmed” Bem’s result by replicating his data. (See the curve in Figure 2 of Wagenmakers et al., 2012, p. 636; Simmons et al., 2011 also illustrate this issue.) Given the small effect size *g* = 3.1% as a theoretical specification of prior results (50% against 53.1%, according to Bem, 2011, p. 409, experiment 1), one should therefore construct a sufficiently strong test-condition to obtain data of sufficient induction quality.^6^

Neyman–Pearson theory defines the necessary sample size (or number of observations, subjects, sessions, etc.) to firmly decide between two hypotheses with a difference of *g* = 3.1%. Since this is a test *against* the H_1_, we should treat both errors equally, for instance by setting α = β = 0.05. It follows that, for the H_0_ to be accepted, it must be 19 times (0.95/0.05) more probable than the H_1_. The necessary sample size (for a proportion difference measured against a theoretical constant of 0.50) then comes to *n* = 2829 (see Cohen, 1977, p. 169). Comparing this tall figure to the *n* = 200 that Wagenmakers et al. (2012) report should make clear why one cannot trust their result.

A well-suited approximation of the necessary sample size, n, given specified errors and a postulated effect size of mean differences, d, is (2(*z*_(1-α)_+ *z*_(1-β)_)^2^)/d^2^= n, where *z*_(1-α)_ and *z*_(1-β)_ increase provided α and β decrease, with *z* taking values greater than 1, and *d* mostly remaining below 1. Given acceptable errors, a very small d (or g) thus generates a large n. So to achieve reasonable certainty under specified errors, we may incur an extremely large number of data points.

The main reason for the large n is the small difference between the two rivaling hypotheses and our rigor in controlling both errors. Therefore, it does not suffice to publish the testing-strategy before and a stopping-rule after data inspection (Nosek and Bar-Anan, 2012; Nosek et al., 2012, 2015). Similarly, though sequential testing is a less problematic way of inflating the α-error than “double-dipping” (Kriegeskorte et al., 2009), it necessarily inflates the β-error, given that we hold effects constant. So it increases the chance of not detecting a true difference, making it improbable to replicate data in independent studies.

### Gauging the Psi-effect

In the case of replicating the psi-hypothesis, the β-error was at least β = 0.58, given *n* = 200, α = 0.05 (one-sided), and *g* = 0.05. Though this is a slightly higher value than was in fact observed, it still qualifies as a small effect (see Cohen, 1977, p. 155). In fact, G^∗^Power software (Faul et al., 2007) estimates an even larger error of β = 0.64. At any rate, the large β-error renders Wagenmakers and colleagues’ test-condition unacceptable.

Based on extant psi-studies, Bem had formulated a H_1_ of *d* = 0.25. (Bem prefers *t*-tests and more “classical” effect size measures over a binomial test; though results are stated in percentages, the output of both kinds of tests is equivalent.) Having specified d, we can thus calculate the error probabilities: for α = 0.05 (one-sided), *d* = 0.25 and *n* = 100, we find β-error = 0.18. Now setting α = β = 0.05, the necessary sample is *n* = 175, smaller than immediately above because d now is comparably large. (*n* = 175 is about half the sample the above formula approximates, since a difference between a constant and an empirical mean is evaluated.)^7^ The Bayes-factor thus registers some 22 times in favor of a H_1_ postulating *d* = 0.25. On a Bayesian view, this is a clear corroboration of the H_1_ over the H_0_.

Though the critical value (1–β-error)/α-error = 0.80/0.05 = 16 has now been surpassed, upon inspecting induction quality it transpires that the result is insufficiently stable. To explain, some of Bem’s trials sought to induce arousal by displaying erotic and non-erotic pictures in random order, measured the degree of arousal before displaying a picture-type, and interpreted heightened arousal *before* showing an erotic picture as evidence of precognition. (This suffices to interpret the set-up as including a control group of sorts.) The observed effect was *d* = 0.19 with *n* = 100 in both samples (erotic vs. non-erotic). Comparing this with the hypotheses *d* = 0.00 and *d* = 0.25, however, a Bayes-factor of 2.18 is now too low. So Bem’s first experiment indeed “discovers” a deviation from random (given α = 0.05, one-sided, and 1–β = 0.82). But the effect isn’t stable (i.e., its reproducibility is insufficiently probable), particularly given that both hypotheses had been specified by recourse to meta-analytical results.

This goes to show that a Bayes-factor may well be extremely large although the effect is not trustworthy. Indeed, if we interpret data from the display of non-erotic pictures as a control group—as we should, because data from the display of erotic pictures did not significantly deviate from random—then a likelihood-ratio of 2.18 (i.e., a logarithm of 0.34) is hardly any evidence for a psi-effect, even if we ignore its low replication probability. Rather, a firm decision under sufficient induction quality requires another sample of *n* = 75 (see below).

### Meta-analyses of Additional Replication Attempts

Bem’s (2011) thought-provoking research has meanwhile initiated something *like* a research program. But we cannot elucidate its contradictory results by relying on *either* frequentist or Bayesian approaches to statistical inference. It should therefore be of interest to clarify the psi-debate by integrating both approaches.

Galak et al. (2012) and Bem et al. (2016) have conducted two independent meta-analyses of psi-studies. Their *combination* in fact yields the necessary sample size. The first study concludes negatively:

“Across seven experiments (*N* = 3,298), we replicate the procedure of experiments 8 and 9 from Bem (2011), which had originally demonstrated retroactive facilitation of recall. *We failed to replicate that finding*. We further conduct a meta-analysis of all replication attempts of these experiments and find that the average effect size (*d* = 0.04) is not different from 0” (Galak et al., 2012, p. 933; *italics added*).

But the second meta-analysis arrives at a quite different result:

“We here report a meta-analysis of 90 experiments from 33 laboratories in 14 countries which yielded an overall effect greater than six sigma, *z* = 6.40, *p* = 1.2 × 10^-10^ with an effect size (Hedges’ g) of 0.09. A Bayesian analysis yielded a Bayes Factor [BF] of 5.1 × 10^9^, greatly exceeding the criterion value of 100 for ‘decisive evidence’ in support of the experimental hypothesis. When Bem’s experiments are excluded the combined effect size for replications by independent investigators is 0.06, *z* = 4.16, *p* = 1.1 × 10^-5^, and the BF value is 3.853, again exceeding the criterion of ‘decisive evidence.’ […] *P*-curve analysis, a recently introduced statistical technique, estimates the true effect size of the experiments to be 0.20 for the complete database and 0.24 for the independent replications” (Bem et al., 2016, p. 1).

To prepare for a critical discussion, consider that both meta-analyses sought to discover a non-random effect, but neither *tested* the psi-hypothesis in the sense of gauging *L*(H|D); effect sizes are heterogeneous, suggesting that uncontrolled influences are at play; Bem’s own studies report larger effects than their independent replications, suggesting a self-fulfilling prophecy; Bayes-*t*-tests, as we saw, depend on the prior distribution and different priors can lead to contradictory results; most studies included in these meta-analyses are individually underpowered.

Of course, to simply aggregate various underpowered studies will not yield a trustworthy inductive basis. After all, almost all mean differences become statistically significant if we arbitrarily divide a sufficiently large sample (*n* ≥ 60.000) into two subgroups (Bakan, 1966). So provided that only the H_0_ is specified, we can almost always obtain a non-random result by increasing the sample. [This contrasts with the methodology of physics (Meehl, 1967), for instance, where a theoretical parameter is fixed and increasing the sample size eventually *disproves* a theory.]

In view of the more than 90 psi-studies that both meta-analyses reviewed, researchers did particularly consider the point-hypotheses *d* = 0.00 (random, H_0_) and *d* = 0.25 (specified, H_1_). (Bem assumed the latter *d*-value to plan studies with power = 0.80, after “predicting” *d* = 0.24 by analyzing independent replications; see Bem et al., 2016.) Since this “research program” requires amendment before it can successfully address the challenges the replicability crisis has made apparent, we now exemplify the inference strategy a *genuine* psi-research program would pursue.

Among the 90 psi-studies, we consider most trustworthy those that arose independently of Bem’s research group, that are classified as exact replications, and that are peer-reviewed (These admittedly rigorous criteria leave but nine of the studies listed in Table A1 in Bem et al., 2016, p. 7, Dataset S1).

Our first critical question must pertain to induction quality: given α = β = 0.05 and *d* = 0.25 (see above), the necessary sample is *n* = 175. The total sample from the nine studies comes to *n* = 520. Since this far exceeds our requirements, we can in fact assess corroboration quality more severely. Given α = β and *n* = 520, the critical value to corroborate the H_1_ now is (1–β-error)/α-error = 0.998/0.002 = 499 (or log 2.70), and correspondingly for the H_0_, i.e., (1 - α-error)/β-error. Adding the log-likelihood-ratios for the H_0_ and subtracting those for the H_1_ then yields 5.13. This value is much higher than 2.70, making it 135,000 times more probable that data inductively support the H_0_, rather than a psi-effect of size *d* = 0.25.

When we gauge the average effect size of our nine studies (weighed by the sample size), moreover, maximum likelihood estimation yields *d* = 0.07 as a psi-effect that is 3.64 times more probable than a random effect. Rather than a final verdict on the true hypothesis, of course, this provides but a relative corroboration of one hypothesis against another. A maximum likelihood estimate that registers so close to the hypothetical parameter, however, fails to provide *any* hint for further research.

The H_0_ is now much better corroborated than the psi-hypothesis. Of course, this does not falsify a psi-hypothesis postulating a *yet smaller* effect. It is nevertheless reasonable to reject the psi hypothesis. After all, to theoretically explain an effect only becomes more difficult the smaller it is. But again, one cannot be certain.^8^

### Summary

Our discussion shows that a *firm and transparent* decision between two specified hypotheses requires knowing the effect size and selecting tolerable α- and β-errors. We also saw how this determines the necessary sample size. Specifically, the smaller an effect is the larger is the necessary sample. Theories that predict small effects should therefore be confronted with much larger samples than is typical. Conversely, the small effects and samples that top-tier journals often report let few published effects count as probably replicable. Indeed, the average test-power reported in Bakker et al. (2012) “predicts” the lower bound (36%) of the average replication rate reported by Open Science Collaboration (2015).

Since many studies avoid specifying the alternative hypothesis, researchers may at best *hope* to find a significant effect among their results. This partially explains why they sometimes “torture” data until significance is achieved. As we saw, such results frequently surface as allegedly important effects, that is, as *genuine* discoveries (see Fanelli and Glänzel, 2013; Witte and Strohmeier, 2013). Predictably, top-tier journals regularly publish studies that fail to report probably replicable discoveries, namely when their test-power is too low (<0.80 or <0.95) to safely reject the H_0_ (Bakker et al., 2012; Francis, 2012; Open Science Collaboration, 2015; Etz and Vandekerckhove, 2016).

With these insufficiently designed studies as evidence for a goal-conflict between psychologists and their field (Witte, 2005; Eriksson and Simpson, 2013), we go on to show how research programs improve the *status quo*.

## Research Programs

### Four Developmental Steps

This section outlines the four steps a progressive research program takes in order to improve empirical knowledge (Hacking, 1978; Lakatos, 1978; Larvor, 1998; Motterlini, 2002). Adopting such programs is a natural consequence of recognizing the precisification of hypotheses as a necessary condition to obtain trustworthy results, and of accepting that prior (fallible) knowledge informs future research.

### Step One: Ideas without Controlled Observation

The first step involves an idea or intuition, perhaps acquired in what C.S. Peirce called *retroduction*. Interesting in itself, *having* that intuition is less relevant for developing a scientific construct. Though an intuition is by definition not based on conscious observation, to be further explored it must nonetheless sufficiently impress us. For instance, we might seek hints in subjective experience, theoretical observation, or collegial discussion. Once we are convinced that the idea *is* relevant, we can engage with it systematically, perhaps moving from material at hand to thought-experiments, computer simulations, or an “idea-paper” (*sans* significance tests, etc.). Importantly, such activities are possible without collecting data.

### Step Two: Devising an Empirical Set-up

The second step establishes our idea with a method, so that it may potentially count as a *genuine* discovery. This requires controlling an empirical set-up in order to “observe” the phenomenon. But we saw that observations may mislead because of random effects and sampling- or measurement-error. So for a discovery to be established, the phenomenon must significantly deviate from random. After all, random variation lacks *specific* semantic content and so does not explain anything but itself.

We also saw that proofs under probabilistic variation are based on inferential statistics that consider the observation’s *p*-value given the H_0_. The classical claim ascribed to Fisher is that a small *p-*value reflects a rare enough event in a random model. (At this step, we cannot properly call the search for small *p*-values ‘p-hacking,’ an often observed strategy after having obtained data.) As a consequence, a “large p” (*p* > 0.05) signals our failure to measure a significant deviation from random. This *prima facie* suggests complicity with a random model. But a large p is expected, of course, if the set-up is insufficiently sensitive to detect an effect that nevertheless *is* present. So it would be naïve to treat the absence of a statistically significant deviation from random as conclusive evidence for the effect’s absence.

This sensitivity caveat calls on us to adjust our logic of decision-making. After all, statistically *insignificant* deviations from random may still signal epistemic value. Historically, that insight led Neyman-Pearson-test-theory to provide us also with an estimate of the β-error. This should be sufficiently small for the chance of not detecting a true effect to be low. In other words, the set-up’s sensitivity should be sufficiently high.

A set-up will generally be *optimal* if evidence features the smallest possible *p*-value given the random model, on one hand, and the largest probability of registering each true deviation from random using a minimum number of observations, on the other. Moreover, an optimal test should be unbiased, so that we can almost certainly detect a true effect as we increase the number of observations. In short, we should select the *most powerful* test-condition. Test-power considerations thus inform how we gauge the relative inductive support that data provide for a content-free H_0_, compared to the support that the same data provided for a (one- or two-sided) H_1_ of substantial content.

At this point, however, rather than manage a point-hypothesis, we still deal with a vague H_1_. Moreover, we know induction quality of data but *partially* as long as we merely hold the α-error constant, but not the β-error. As a consequence, data may now *seem* to corroborate or falsify a hypothesis, but they may not be replicable. Lastly, corroboration and falsification equally depend on the distribution of possible data. So corroboration quality is at best *diffuse*.

Even if data should now “eliminate” the H_0_, as it were, a genuine *bundle* of substantial alternative H_1_-hypotheses remains to be eliminated. Since each such H_1_-hypothesis postulates a distinct effect size, the whole bundle provides mutually exclusive explanatory candidates. Each such H_1_ therefore associates to a distinct likelihood-ratio as its corroboration measure. So the unsolved problem of validating induction merely let data *hint* at the correct theoretical parameter, but data alone cannot determine it. Instead, it is *our* having further specified this parameter that eventually lets data decide firmly between any two such point-hypotheses.

### Step Three: Replication and Meta-analysis

As we achieve replication-success several times, we can give a better size-estimate of a significant effect. But this estimate will be unbiased only if we also base it on (often unpublished) statistically non-significant results (Sterling, 1959; Scargle, 2000; Schonemann and Scargle, 2008; Ferguson and Heene, 2012). Moreover, an effect size that has remained heterogeneous across several studies should eventually be differentiated from its test-condition. In fact, this is the *genuine* purpose of a meta-analysis. But extant analyses are heavily biased toward published results. Despite various bias detection-tools, such analyses therefore often present a skewed picture of all available data, and hence exaggerate the true effect size (Francis, 2012).

When we reproduce an effect, we should therefore correct a plain induction over prior findings by more theoretical strategies (see, e.g., Witte, 1994, 1996b, 2005). This includes data-inspection and -reconstruction by means of a theory with a mathematical core that predicts quantitative results while retaining an adequate connection to data. Data reconstruction thus becomes a stepping stone to formulating point-hypotheses.

The forgoing exhausts the relevant discovery context activities. Subsequent work should be guided by specifying effect sizes as point-hypotheses, and by testing them against *new* data that arise as retrodictions or predictions.

### Step Four: Precisification of Effect Sizes and Theoretical Construction

As we saw, we can quantitatively assess the quality of an empirical set-up only *after* a point-hypothesis is available. Subsequently, a set-up’s induction quality can serve as a criterion to probabilistically corroborate, or falsify, a theoretical construct. So the fourth step directly concerns formulating precise point-hypotheses.

A clear indicator that we have reached the justification context is to inspect likelihoods of hypotheses given data, *L*(H|D), rather than probabilities of data given hypotheses, *P*(D,H). Whether precision-gains then arise from a parameter-estimation or by combining significant results (Witte and Zenker, 2016a,b, 2017), here we either induce over empirical results, or we perform a quantitative reanalysis. This marks the onset of an explanatory construction (Witte, 1996b; Witte and Heitkamp, 2006). As a rule, if we have corroborated an effect size, we should next provide a theoretical (semantic) explanation.

**FIGURE 1:**
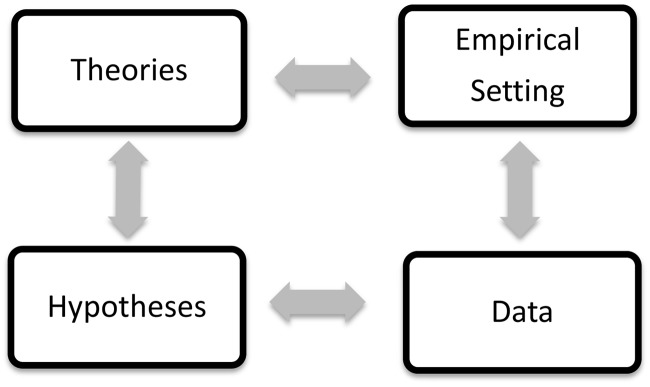
Only stable data (“phenomena”) can be meaningfully subsumed under empirical hypotheses (bottom arrow); such hypotheses can be systematized into theories (left arrow) that retrodict old and predict new data; this may lead to constructing empirical set-ups (top arrow) for new phenomena (right arrow), which potentially improve extant theories.

Generating such explanations takes time, of course, and often incurs unforeseen problems. Indeed, it need not succeed. With Lakatos (1978), we consider a research program *progressive* as long as a theoretical construction or its core-preserving modification generate predictions that are at least *partially* corroborated by new data of sufficient induction quality. Any such construction may therefore lead to further discoveries, e.g., in the form of observations that deviate from random. So it remains overly simple to treat theory-development as a linear process (see **Figure 1**). But that scientific progress grounds in fallible knowledge of an original phenomenon deserves acceptance.

A basic principle is to continue the precisification of hypotheses, since this improves both induction quality (from *unknown* to *known to be probably reproducible*) as well as corroboration quality (from *diffuse* to *precise*). A progressive research program thus shifts the focus, away from vague justification against the H_0_, toward precise justification of a point-specified H_1_. That said, HARKing and double dipping (Kerr, 1998; Kriegeskorte et al., 2009; Simmons et al., 2011) remain non-cogent justifications because each so generated result must lead to a confirmation.

## Move Over, Please!

Statistical inference is a tool to corroborate theoretical assumptions rather than a machinery to generate theoretically grounded empirical knowledge—which an empirical science must justify with trustworthy data. Therefore, a loose discovery under vague hypotheses (based on sample-estimations) can never lead to a firmly corroborated theoretical assumption. In fact, to even only disconfirm a precise assumption is already far more informative than to never achieve adequate precision.

Considering the scarcity of similarly well-hardened knowledge in current psychological research, the field’s progress demands justification context-activities (Ioannidis, 2012). Indeed, as long as the field as a whole remains in the discovery context, it is doubtful that lenient reviewers, full data disclosure, or removing the publication-bottleneck, etc. even address the true challenges. Instead, we would presumably see meta-analyses lead to meta-meta-analyses, which however cannot serve a progressive research program (Schmidt et al., 2009; Cafri et al., 2010; Stegenga, 2011; Chan and Arvey, 2012; Ferguson and Heene, 2012; Mitchell, 2012). Rather, if a meta-analysis discovers an effect, one should seek to confirm a precisified version thereof by successfully predicting it in *new* data.^9^

The likelihood ratio, as we saw, is the measure of corroboration quality. For a tolerable error-range, reasonable certainty that a H_1_ is justified thus requires that its likelihood-ratio exceeds a conventional threshold. We can compare the error terms from Neyman-Pearson-theory to Jeffrey’s (1961) qualitative classification: α = β = 0.05 translates into a likelihood-ratio of 19 (“strong evidence”) and α = β = 0.01 into a ratio of 99 (“very strong evidence,” “nearly extreme evidence”). Further, provided that α = β ≤ 0.01, the degree of corroboration for the supported hypothesis will in the long run approach the test-power value (Wald, 1947).

As a summary, we now list six increasingly important results of a research program and their measures. The last result corroborates a point-hypothesis.

(1) *preliminary discovery*: α-error or merely a *p*-value (to establish that data are non-random)
(2) *substantial discovery*: α and 1–β-error (to gauge replicability based on a specific effect size and a particular sample size)
(3) *preliminary falsification* of the H_0_: *L*(d_H1_ > 0)/*L*(d_H0_ = 0) (to establish a H_1_ that deviates in one direction from the H_0_ as more likely than the random parameter *d* = 0)
(4) *substantial falsification* of the H_0_: *L*(d_H1_ > Δ)/*L*(d_H0_ = 0), where Δ is the theoretical minimum effect size value (to establish a H_1_ as non-random and also exceeding Δ)
(5) *preliminary verification* of the H_1_: *L*(d_H1_ = Δ)/*L*(d_H0_ = 0) (to corroborate the theoretical parameter Δ against the random parameter)
(6) *substantial verification* of the H_1_: *L*(d_emp, H1_)/L(d_H2_ = Δ) < 4, given approximately normally distributed data, where d_emp_ is the empirical effect size^10^ (to indirectly corroborate Δ against the maximum-likelihood estimate of empirical data, d_emp_).

The justification context starts with line (3). So the unsophisticated application of NHST sees large parts of empirical psychological research “stuck” with making preliminary or substantial discoveries. Notice, too, that the laudable proposal by Benjamin et al. (2017) to drastically lower the α-error merely addresses what shall count as a preliminary discovery. But it leaves unaddressed how we establish a substantial discovery as well as the justification context as a whole.

## Precise Theoretical Constructs?

Allow us to briefly speculate why the goal conflict between generating statistically significant results and generating trustworthy fallible knowledge has played out in favor of the former goal. We saw that trustworthy data is primarily relevant toward developing and testing more refined theoretical constructs. But theory-construction is rarely taught at universities (Gigerenzer, 2010). So the replicability crisis also showcases our inability to in fact erect the constructs that statistically significant effects should lead to (Ellemers, 2013; Klein, 2014).

To give but two examples, the 51 theories collected in a recent social psychology handbook (van Lange et al., 2012) cannot achieve equally precise predictions as those that a random model offers. But not only should we base a fair decision between two hypotheses on data that are known to be probably replicable; we should also require *parity of precision* (Witte and Kaufman, 1997). By contrast, a two volume edition on small group behavior (Witte and Davis, 1996) contains eleven theories that are sufficiently elaborated to predict precise effects, while another ten theories make vague predictions. So precise constructions *are* possible, yet they are rare. Therefore, calling the replicability crisis “home-made” does not directly implicate career aspiration or resource shortage. Rather, ignorance of how one constructs a theory seems to be an important intermediate factor.

The crisis will probably seem insurmountable to those who disbelieve that a developing research program is even possible. Alas, might one not first ask to show us a *failed* research program? Indeed, would not a research program culture need to have been established, and to have broadly failed, too? If so, then an urgent challenge is to coordinate our research away from the individualistically organized but statistically underpowered short-term efforts that have produced the crises, toward jointly managed and well-powered long-term research programs.

Of course, it takes more than writing papers. Indeed, funding-, career- and incentive-structures may also need to change, including how empirical psychologists understand their field. It may even require that larger groups break with what most do quasi-habitually. This, perhaps, would establish what our paper only described.

## Conclusion

The replicability crisis in psychology is in large part a consequence of applying an unsophisticated version of NHST. This praxis merely generates theoretically disconnected “one-off” discoveries—that is, effects which deviate statistically significantly from a random model. Parallel to it runs the interpretative or rhetorical praxis of publishing such effects as scientifically important results, rather than as the parameter estimations they are. The former praxis fails to maximize the utility of a sophisticated version of NHST, which nevertheless remains a useful and elegant approach to gauging *P*(D,H). The latter praxis regularly over-reports *P*(D,H) as *L*(H|D), which even the most sophisticated application of NHST, however, cannot warrant. Both praxes may plausibly have arisen from not fully understanding the limits of NHST.

The field has thus “discovered” many small effects. But whenever empirical studies remain underpowered, because they comprise too few data-points, research efforts remain in the discovery context. Here, vague effect sizes and vague alternative hypotheses are normal. But such results fail to convince as trustworthy effects that deserve theoretical explanation. By contrast, a clear indicator that research has shifted to the justification context is a likelihood-ratio-based decision regarding a precisified effect size that is expectable in *new* data of known induction quality (entailing a Bayes-factor for fixed hypotheses).

**FIGURE 2:**
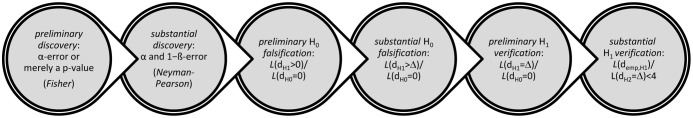
Salient results of a research program.

This requires a diachronic notion of the research process—a developing *research program*—that adapts statistical inference methods to prior knowledge. Schematically (**Figure 2**), we start with *p*-values (Fisher), move on to an optimal test against a random-model (Neyman–Pearson with α- and 1-β-error), accompanied by parameter estimation via meta-analysis, to achieve—entering the justification context—a corroboration of a theoretically specified effect size based on stable data of known induction quality (under tolerable errors), against a random model or against another point-specified hypothesis.

In particular, we should deduce from a theory a specified effect size that goes beyond the simple assumption of a minimal effect. A quantitative specification without theoretical explanation may well be a first step (assuming known induction quality and precise corroboration quality). But a successful research program must derive a prediction from a theoretical model *and* provide an explanation of the effect’s magnitude. For instance, psi-research would only gain from such an explanation.

Since many theories only offer vague predictions, moreover, the lack of confidence among psychologists might at least partially result from failed theory-development. But the replicability crisis itself is narrowly owed to underpowered studies. Making headway takes researchers who join forces and resources, who coordinate research efforts under a long-term perspective, and who adapt statistical inference methods to prior knowledge. To this end, we have also seen a strategy for combining data from various studies that avoids the pitfalls of extant meta-analyses. It is in the *integrated* long run, then, that empirical psychology may improve.

## Author Contributions

Both authors have jointly drafted this manuscript; the conceptual part of this work originates with EW; FZ supplied additional explanatory material and edited the manuscript.

## Conflict of Interest Statement

The authors declare that the research was conducted in the absence of any commercial or financial relationships that could be construed as a potential conflict of interest.

## Footnotes

1 In fact, OSC reported an average replicability-rate of some 36%. Worse still, a Bayesian approach finds clear and consistent results in merely 11% of 72 reanalyzed datasets (Etz and Vandekerckhove, 2016). Though Gilbert et al. (2016, p. 1037-a) submit that “[i]f OSC (Open Science Collaboration) had limited their analyses to endorsed studies, they would have found 59.7% [95% confidence interval: 47.5, 70.9%] were replicated successfully," it is clear that even 59.7% is insufficient to regain trust.

2 As a recent survey indicates, psychologists are open to milder changes (Fuchs et al., 2012). The accepted rules of best research practice, for instance, shall not be turned into binding publication conditions. Moreover, 84% among respondents find that reviewers should be more tolerant of imperfections in what their peers submit for publication (ibid., p. 640). Additional proposals include intensifying communication, pre-registering hypotheses, and exchanging data- and design-characteristics (e.g., Nosek and Bar-Anan, 2012; Nosek et al., 2015).

3 Introduced by Reichenbach (1938), the DJ-distinction has been contended from diverse perspectives, notably since the reception of Kuhn (1970). Extant discussion of the distinction’s multiple versions questions the separability of both contexts on temporal, methodological, goal- or question-related criteria. See Schickore and Steinle (2006) and references provided there.

4 The inferential strategy generating this question entails an inductive transition. After all, by formulating a specific non-random hypothesis the probability model, as it were, transforms “observational” data into a quasi-theoretical hypothesis. So we run into the unsolved issue of justifying induction as a *valid* inference (see below).

5 This praxis is not restricted to NHST but also implicates proponents of Bayes-factor testing. Verhagen and Wagenmakers (2014, p. 1461), for instance, state that “[t]he problem with this analysis [likelihood-testing] is that the exact alternative effect size δ_a_ is never known beforehand. In Bayesian statistics, this uncertainty about δ is addressed by assigning it a prior distribution.” Indeed, “[t]he major drawback of this procedure is that it is based on a point estimate, thereby ignoring the precision with which the effect size is estimated” (ibid., p. 1463). Similarly, “[w]e assumed that the alternative was at a single point. This assumption, however, is too restrictive to be practical” (Rouder et al., 2009, p. 229).

6 We use ‘g’ to express the effect size as the difference between a stipulated proportion of 0.50 and an observed proportion, using the sign test; ‘d’ denotes a difference between numerical means (Cohen, 1977). Notice that when errors and likelihoods are calculated via a normal curve approximation of the effect size, the model remains constant under different proof distributions.

7 Here restricting the focus to a two sample (Neyman–Pearson) *t*-test with α- and β-errors, a draft on the one sample *t*-test and the negative consequences of various “saving-strategies” to reduce the sample size can be obtained from the authors.

8 It is natural to object that very few people may in fact command psi-abilities. This might seem to explain why Bem can measure only a small effect. However, this explanation-sketch presupposes that we could (somehow) aggregate effect sizes from individually underpowered studies into a “pooled” effect size. Instead, what we can safely aggregate are log-likelihoods (see Witte and Zenker, 2016a,b, 2017). The explanation-sketch might nevertheless lead to a new hypothesis, namely: can we reliably separate subjects into those who command and those who lack psi-abilities? If so, we should next test among the former group if the psi-effect is stable. At any rate, research addressing such hypotheses qualifies as a discovery context activity.

9 Such research clearly differs from parameter-estimation because it can bury “undead” theories. But this is impossible given how standard methods are mostly used today (Ferguson and Heene, 2012; Eriksson and Simpson, 2013).

10 If the likelihood value of the theoretical parameter does *not* fall outside of the 95%-interval placed around the maximum-likelihood-estimate, then we view the theoretical parameter as substantially verified, and thus corroborated. After all, both the empirical result and the theoretical assumption now lie within an acceptable interval. The corroboration threshold is given by the ratio of the two likelihood-values, i.e., the maximum ordinate of the normal curve (0.3989) and the ordinate at the 95%-interval (0.10). To good approximation, this yields 4.
